# A Mixed-Methods Case Report on Oral Health Changes and Patient Perceptions and Experiences Following Treatment at the One Smile Research Program: A 2-Year Follow-Up

**DOI:** 10.3390/clinpract15080136

**Published:** 2025-07-23

**Authors:** Mona Abdelrehim, ZhuZhen (Hellen) Huang, Christiana Martine, Imon Pal, Kamini Kaura, Anuj Aggarwal, Sonica Singhal

**Affiliations:** 1 Faculty of Dentistry, University of Toronto, Toronto, ON M5G 1G6, Canada; zhuzhen.huang@mail.utoronto.ca (Z.H.); christiana.martine@utoronto.ca (C.M.); imon.pal@utoronto.ca (I.P.); kamini.kaura@alumni.utoronto.ca (K.K.); anuj.aggarwal@dentistry.utoronto.ca (A.A.); sonica.singhal@dentistry.utoronto.ca (S.S.); 2 Public Health Ontario, Toronto, ON M5G 1M1, Canada

**Keywords:** dental care, health services accessibility, patient satisfaction

## Abstract

**Background**: In Canada, despite universal healthcare coverage, dental care remains predominantly privately financed, creating financial barriers that prevent many from accessing essential services. This case study is part of a larger initiative, the One Smile Research program, which evaluates the impact of cost-free dental care on the oral health and overall well-being of individuals who have been unable to access dental services in the past two years due to financial constraints. Participants in the program receive necessary dental care and attend follow-up appointments to assess the long-term effects of continuous cost-free care. **Clinical Case**: This mixed-methods case report focuses on a 26-year-old male participant and integrates a qualitative semi-structured interview with clinical and self-reported data, providing an in-depth understanding of his experiences. **Results**: Clinical outcomes demonstrated the effectiveness of the provided dental treatments, while self-reported measures indicated improved oral health, satisfaction with dental appearance, enhanced psychosocial well-being, increased self-esteem, reduced dental anxiety, and better oral hygiene habits. The qualitative interview identified three key themes reflecting positive experiences with the program: ease of admission, staff kindness, and overall well-being improvement. The integration of both quantitative and qualitative analyses revealed significant advancements in both objective and subjective measures, particularly regarding overall well-being. **Conclusions**: The continuity of cost-free dental care effectively addressed the participant’s oral health and overall well-being, with most benefits sustained even at the two-year follow-up. These individual-level outcomes offer preliminary insight into the potential advantages of universal dental coverage within the Canadian healthcare system.

## 1. Introduction

The World Dental Federation (FDI) describes oral health as multifaceted. It encompasses conditions of the oral structures and diseases, such as dental caries, periodontitis, and oral cancer. It also involves physiological functions associated with these structures, such as speaking, smiling, smelling, tasting, touching, chewing, and swallowing without pain or discomfort. Furthermore, it includes psychosocial functions, allowing individuals to express emotions through facial expressions with confidence and without embarrassment [1]. Given this comprehensive understanding, oral health care should adopt a holistic approach that addresses both normative and subjective needs.

In Canada, while healthcare is publicly funded, dental care remains a personal responsibility for most Canadians, with the exception of medically necessary surgical-dental services delivered in publicly funded hospitals [2]. Approximately 33% of Canadians lack any form of dental care coverage and pay fully out-of-pocket for services [3]. Even among those who have dental care coverage, co-payments, which account for 20% to 50% of treatment costs, can deter individuals from seeking care [4]. Furthermore, trends in cost barriers to dental care have risen over the past 15 years, negatively impacting oral health [5]. Poor oral health is more common among individuals who lack access to regular dental care, underscoring the critical role of cost as a barrier [6].

The inability to access oral health care is a determinant of poor oral health; however, it remains unclear whether continuous access to dental care without financial barriers improves and maintains oral health outcomes. To assess the impact of cost-free dental care on enhancing and sustaining individuals’ oral health, the “One Smile Research” program, a single-arm, repeated measures clinical trial, was launched at the Faculty of Dentistry, University of Toronto. The primary eligibility criterion is a self-attested declaration of inability to access dental care within the last two years due to affordability issues. Additional eligibility requirements included residency in the City of Toronto or the Greater Toronto Area (GTA), a valid mailing address, English proficiency, and the ability to provide verbal and written informed consent. There is no age restriction in the study.

The research project, which received approval from the Research Ethics Board at the University of Toronto (Ethics Board #39888) and commenced in November 2021, was launched to advance the policy agenda for universal dental care coverage in Canada. As an action research initiative, the study seeks transformative change by simultaneously conducting research and taking action, using a holistic, multi-method approach to problem-solving [7].

In addition to the quantitative analyses of participants, an optional qualitative component was added to gain a more comprehensive understanding of the barriers faced by the disadvantaged individuals. This component aimed to capture nuances not captured during the quantitative analysis alone and enhance the trustworthiness of findings through triangulation [8]. By adopting a mixed-methods approach, the study seeks to amplify the patient’s voice—an often-overlooked perspective in public health program design—and to provide insight into how such care is experienced, valued, and internalized by the recipient. This work contributes to a more patient-centered understanding of the impact of publicly funded dental interventions.

## 2. Case Presentation

### 2.1. Methods

This mixed methods case report is both intrinsic, seeking a deeper understanding of the specific case of interest, and illustrative, providing descriptive and in-depth examples to augment information about a program or policy [9]. This case report features a 26-year-old patient who self-identified as a white male of European descent, born in Canada. At the time of recruitment, the participant was single, had never been married, was unemployed, and held less than a high school diploma. In terms of living arrangements, he lived with one other person in rented accommodation. Financially, the participant had no dental insurance and reported facing barriers to accessing dental care over the past two years. At baseline, the annual household income ranged between $20,000 and $30,000, while the participant’s personal income was $2500.

The participant applied to the One Smile Research program in March 2022. Upon being deemed eligible, the participant provided verbal and written informed consent and completed a medical/dental history form, along with a self-administered survey at the first visit, for a baseline assessment (Supplementary File S1). The survey, hosted on REDCap (Research Electronic Data Capture) at the University of Toronto, utilized closed-ended questions. REDCap is a secure, web-based software platform designed for research data capture. Self-reported oral health was assessed through a combination of global questions (oral health, oral pain, satisfaction with appearance, life stress) and validated questionnaires: the psychosocial impact of oral health and treatment (measured by the Psychosocial Impact of Dental Esthetic Questionnaire, PIDAQ) [10], dental anxiety (measured by the Modified Dental Anxiety Scale, MDAS) [11], self-esteem (measured by the Rosenberg Self-Esteem Scale, RSES) [12], and social functioning (measured by the Social Functioning Scale, SFQ) [13]. The score ranges and interpretation guidelines are outlined in Table 1. Further details about these instruments can be found in the included references.

Following the baseline survey, a dental hygienist conducted an initial oral assessment and took radiographs, which were then reviewed by the study dentist. The clinical examination was performed to assess several key aspects, including dentate status, oral function, masticatory ability, and dental treatment needs (e.g., preventive, endodontic, prosthetic). It also evaluated the presence of prostheses, the type of occlusion, mucosal health, a history of dental decay, and periodontal health. In collaboration with the participant, a dental treatment plan was developed. After completing the necessary treatment and achieving oral health stability (Supplementary File S2), recall appointments were scheduled for 15 days, 6 months, and then annually at 1 and 2 years. These follow-ups included post-treatment self-administered surveys (Supplementary File S3) and clinical assessments.

The qualitative component was informed by a combination of both critical and social constructivist paradigms, which, according to Crotty [14], are inherently compatible in terms of their ontology and epistemology. Qualitative data were collected through a one-time, 30-min semi-structured interview conducted in person at the University of Toronto’s dental clinic, aiming to complement the quantitative component of this study and to provide contextual insights into the dental program. The interview guide (Supplementary File S4) was designed by researchers ZH, IP, and KK around three overarching domains of inquiry to understand patients’ experiences undergoing treatment under the One Smile Research program: access to care, quality of care, and the impact on overall well-being. These domains were not theoretically informed but were created around areas of practical interest and priority for the One Smile staff and researchers. The in-person interview was conducted by researchers KK, MA and IP, audio-recorded, de-identified, and then transcribed verbatim using the Zoom platform. A descriptive thematic analysis—commonly ascribed to be a generic form of qualitative analysis [15]—was conducted following Braun and Clarke’s [16] guidelines, as follows: First, the generated transcripts were proofread by researcher CM against the original audio recording and read multiple times to develop familiarity with the content and a broader understanding of the material. Any preliminary insights were recorded in the form of observational asides. Second, a manual, line-by-line coding approach was conducted inductively by researcher CM. Initial broad categories were identified in the data, and corresponding data extracts were collated accordingly. This iterative process involved continual reference to the primary data to enhance comprehension and ensure consistency in interpretation [15,16]. Third, after the interview transcript was preliminarily coded, initial categories were grouped by researcher CM into potential ‘theme piles.’ Some categories were merged into overarching themes, while others that did not pertain to any newly created theme were either discarded or placed in a miscellaneous category. Fourth, the themes were further refined and reviewed to assess whether they were coherent with the associated data extracts and whether they accurately reflected their core essence. Initial codes, categories, and the refinement of themes were discussed at various stages among researchers CM, MA, and SS to strengthen trustworthiness.

#### 2.1.1. Baseline Exam

At baseline, as per the medical and dental history questionnaire, the patient reported no medication usage or allergies and no history of smoking or alcohol consumption. The patient indicated the presence of dental cavities at the time of enrollment. His oral hygiene practices included brushing 4–5 times a week with no reported flossing. Complaints of halitosis and bleeding during tooth brushing were noted. Additionally, the patient presented with asymptomatic clicking on the left side of his jaw.

During the clinical assessment, the patient reported pain in the lower right quadrant and expressed anxiety about dental treatment. During the initial blood pressure assessment, the patient presented with high blood pressure at the first reading (150/98), followed by a decrease on a second reading (146/87). As the systolic blood pressure was still high on the second reading, the patient was advised to follow up with his physician.

**Figure 1:**
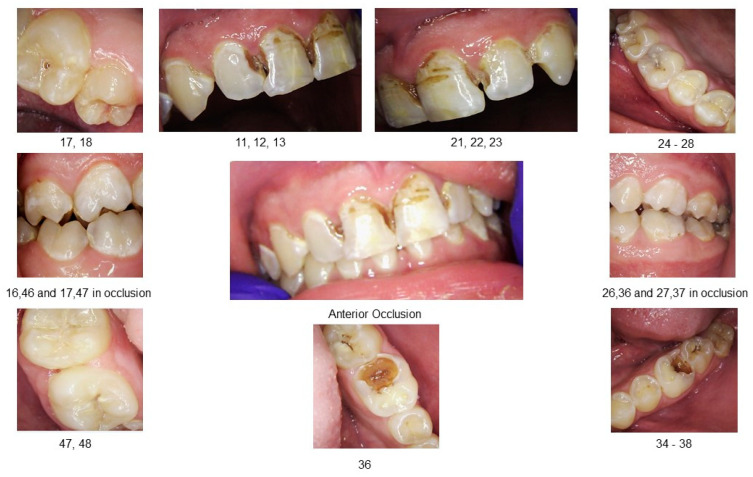
Pre-treatment intraoral photographs.

**Figure 2:**
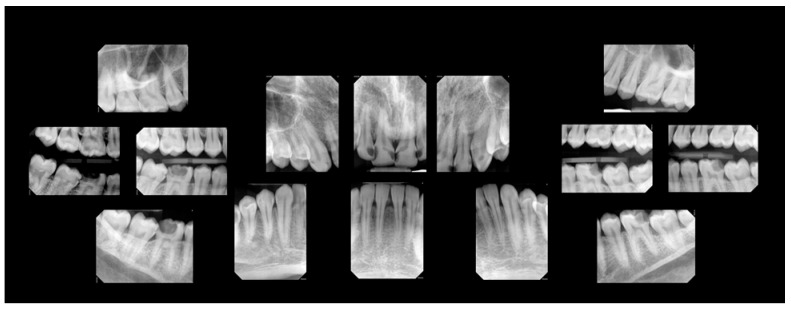
Pre-treatment radiographs.

A comprehensive oral examination, including intraoral photographs and full-mouth radiographs (Figure 1 and Figure 2), was conducted. The examination revealed that the patient had a full set of 32 teeth. Among these, 25 teeth exhibited active decay affecting single and/or multiple surfaces, 6 had incipient carious lesions, and 1 tooth was sound. Fractures were identified on the incisal surfaces of teeth numbered 41, 31, and 32. Furthermore, the distal–occlusal lingual wall of tooth 46 was found to be fractured, and tooth 28 exhibited extrusion.

Among the multiple teeth requiring restoration, teeth 36 and 46 exhibited deep caries. Testing with Endo Ice confirmed their vitality, showing a response within normal limits. The restorability of teeth 36 and 46 was deemed questionable at the time of examination due to the deep lesions. The patient was informed of a conservative treatment approach for these specific teeth, with the possibility of root canal treatment in the future. Generalized moderate sub-gingival and supra-gingival calculus was evident. Periodontal probing was within normal limits (less than 3.0 mm on average). Bleeding on probing was noted in 94% of the examined sites, calculated using the following formula: [number of bleeding sites/(number of teeth X 6 sites per tooth)].

#### 2.1.2. Dental Treatment

**Figure 3:**
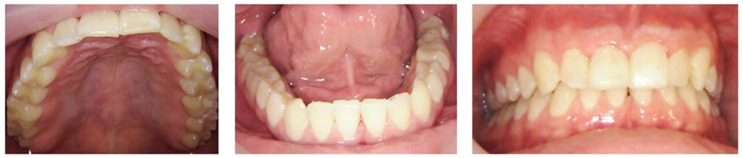
Post-treatment intraoral photographs.

Dental treatment began with oral prophylaxis, including full-mouth scaling, fluoride varnish application (5% sodium fluoride), and oral hygiene instructions. Over eight appointments, restorative treatment was provided under local anesthesia (4% articaine with 1:200,000 epinephrine). After caries excavation, no pulp exposure was observed. Following selective etching using 37% phosphoric acid and the application of universal bond, active carious lesions were restored with composite resin, with or without the application of resin-modified glass ionomer base liner. Occlusion, proximal contacts, and margins were verified, and restorations were finished with finishing burs and polished. Postoperative instructions were provided at each visit (Figure 3). Incipient lesions were noted in the patient to be routinely monitored.

Ten days post-completion of the needed dental treatment, the patient reported discomfort while biting on the lower left side, specifically related to tooth 36. No sensitivity to cold was noted, and upon reassessment, the bite appeared normal. The patient was advised to monitor the tooth as the restoration was deep. Afterwards, the patient achieved oral health stability as per the study criteria and transitioned into the follow-up stage.

At the 15-day and 6-month follow-ups, periodontal probing was conducted, and the patient completed post-treatment questionnaires. At both the one-year and two-year follow-up appointments, radiographs were taken, a recall exam was performed, and full-mouth scaling was completed.

At the one-year follow-up, the patient reported pain in the lower right quadrant. Radiographic evaluation revealed a chronic periapical abscess on tooth 46. Endodontic treatment was performed under local anesthesia (4% articaine with 1:200,000 epinephrine). After access opening, three root canals were located. The canals were irrigated with 5.25% sodium hypochlorite, shaped using endodontic files to primary size, and obturated using gutta-percha cones with sealer. Root canal treatment was completed in a single visit, which addressed the pain.

**Figure 4:**
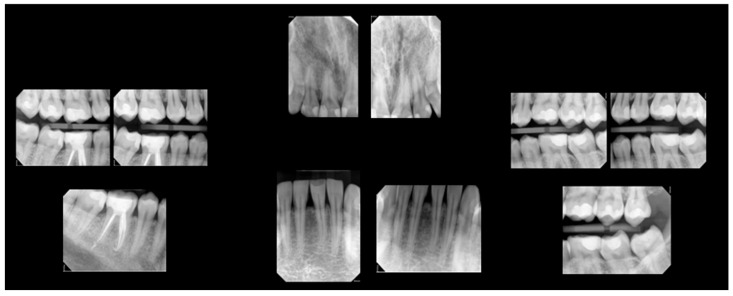
Two-year follow-up radiographs.

By the two-year follow-up, the patient presented with progression of caries on the buccal surfaces of teeth 38 and 48, which were incipient at baseline. Radiographs from this follow-up appointment are represented in Figure 4.

**Figure 5:**
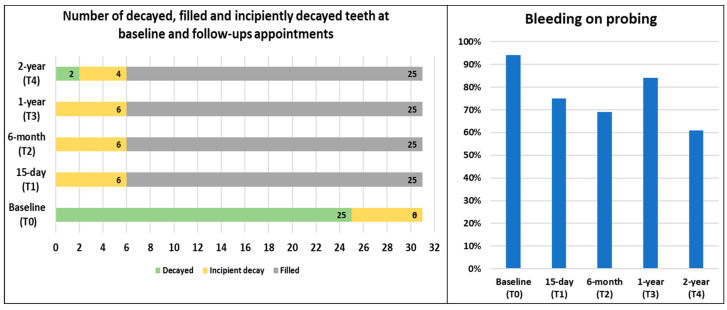
Number of decayed, filled, and incipiently decayed teeth and percentage of sites reporting bleeding on probing at baseline and follow-up appointments.

While the average pocket depth at baseline was in the normal range, there was a slight decrease at both the one-year and two-year follow-ups. Bleeding on probing decreased substantially from 94% at baseline to 61% at the two-year follow-up. Additionally, the patient initially presented with generalized moderate calculus, which improved to generalized mild calculus by the 6-month follow-up and was maintained through the 2-year follow-up. Figure 5 outlines changes in the number of decayed, filled, and incipiently decayed teeth from baseline through each follow-up appointment, as well as the bleeding on probing percentages.

#### 2.1.3. Self-Reported Questionnaire

The patient’s self-reported oral health perception at baseline was poor; however, notable improvements were observed post-treatment at the 15-day and 6-month follow-ups, with the patient rating his oral health as “very good.” However, at the one-year follow-up (T3), the patient rated his oral health as “fair,” likely due to pain and the need for root canal treatment. By the two-year follow-up (T4), the patient again reported his oral health as “very good. There was a marked enhancement in the satisfaction with his dental appearance, which transitioned from “very dissatisfied” before treatment to “satisfied/very satisfied” post-treatment, a positive change sustained through the two-year follow-up.

Regarding oral hygiene practices, the patient initially reported brushing once per day, which increased to twice per day at the 15-day follow-up and then reverted to once per day at the 6-month, 12-month, and 2-year assessments. A noteworthy positive change occurred when the patient incorporated daily flossing into his routine after completing dental treatment, which he maintained for one year; however, he reverted to not flossing by the two-year follow-up.

**Figure 6:**
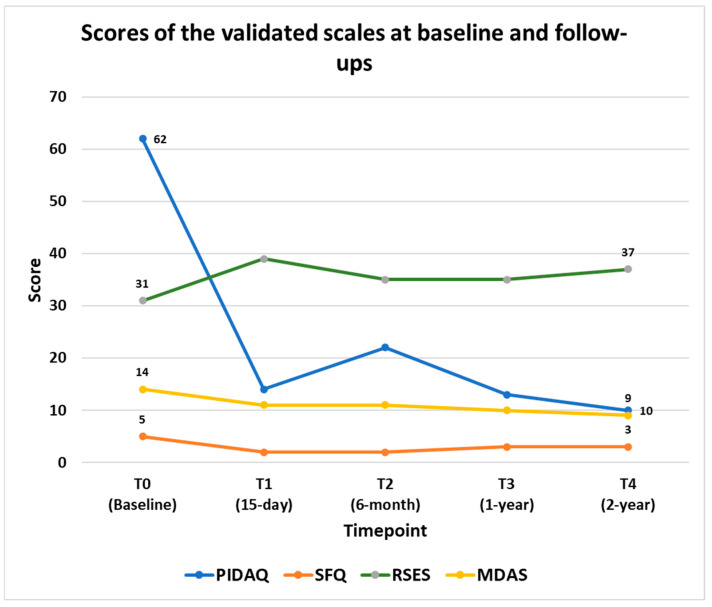
Scores of the validated scales at baseline. 15-day (T1), 6-month (T2), 1-year follow up (T3), and 2-year follow-up (T4).

Psychosocial aspects showed a substantial transformation, with the patient reporting feeling embarrassed because of his dental appearance “fairly often” before treatment and “never” afterwards, a feeling that persisted through the two-year follow-up. In addition, there was a significant decline in the PIDAQ score from 62 at baseline to 10 at the two-year follow-up, highlighting sustained improvements in psychosocial well-being even after 2 years post-treatment (Figure 6).

Self-esteem, as measured by the RSES, improved from 31 at baseline to 39 at the 15-day follow-up, stabilizing at 37 at the 1-year and 2-year follow-ups (Table 1). Similarly, social functioning also showed improvement. According to the SFQ scale, the patient demonstrated good social functioning at baseline with an SFQ score of 5. Following dental treatment, this score improved to 2 at both the 15-day and 6-month follow-ups and to 3 at both the 1-year and 2-year follow-ups (Figure 4).

Dental anxiety, measured using the MDAS scale, decreased from 14 before treatment to 11 at both the 15-day and 6-month follow-ups, further dropping to 10 at the 1-year follow-up and 9 at the 2-year follow-up (Figure 4). In general, before treatment, the patient reported being “slightly anxious or depressed,” which changed to “not anxious or depressed” after one year of treatment; this improvement was sustained at the two-year follow-up. The patient reported increased confidence in meeting new people after receiving dental treatment, a change that persisted through the two-year follow-up. His self-perception about his general health also improved from “poor” before treatment to “very good” after treatment. Additionally, his employment status changed from being initially unemployed at the baseline to part-time employment at both the one-year and two-year follow-ups.

#### 2.1.4. Qualitative Findings

The qualitative analysis yielded three main themes highlighting the patient’s perception of undergoing treatment at the One Smile Research program: the ease of admission, the kindness of the staff, and an improvement in overall well-being.

##### Ease of Admission

After learning about the program through a family member, the participant reported searching for the dental program online and found no difficulty filling out the application. Although he considered the application process straightforward, with a simple roster of questions, he experienced a three-month delay between his initial attempt to register for the dental program and his actual enrollment.

*Just went on Google. Searched up like U of T Green Shield Clinic, I think it was at first. Like went, selected the website and went in. I could not sign up at first because all the spaces were taken, but I had to wait, like three months I think, and then I signed up.*

##### Kindness of the Staff

The participant described the One Smile Research program providers as “knowledgeable,” “helpful,” and “calm.” It is noteworthy that the term “nice” is prevalent and recurrent in the data codes related to the quality of care rendered at the research program. The data suggest that this attribute is not only linked to a pleasant patient–provider interaction and a positive overall patient experience—diametrically opposed to his single experience previously with dental care abroad—but also connected to his ability to better understand and incorporate hygiene routines considered critical for maintaining stability in oral health, such as flossing. Moreover, his perception that “people are nice” may contribute to better acceptance among those seeking public dental services, as illustrated in the following excerpt:

*Where I come from like in Europe, I guess, like the doctors are more rude. Like, I guess would be the term… People [providers] might say things, things that you haven’t done [reprimanding the patient] and stuff like that. In my experience, this is just amazing cause the people [providers at OSP] are too nice… Oh, they were very helpful and nice, right? I mean, I learnt things that I did not know about, of course. Like flossing, for example. Like, [I] never even knew what that was until I got here [dental clinic]… [Patients in need of public dental care] like their incomes are lower. So, obviously, they feel better if I tell them what they get when they sign up and the people are nice, [and] stuff like that.*

##### Improvement in Overall Well-Being

When discussing the impact of receiving dental care at the One Smile Research program, the participant believed that the benefits to his overall well-being outweighed any potential “negatives” during or after treatment. Although he stated at one point in the interview that “there are [were] no negatives,” he later recalled experiencing pain at the beginning of the treatment at the clinic. However, he reflected that this may be expected and a normal occurrence when starting dental treatments. While he reported experiencing “unbearable” pain before registering for the program and mentioned that creating new, healthy habits were “positives” resulting from his treatment at the research program, the most notable impact on his well-being was, undoubtedly, gaining social confidence.

*Like, the positives are, like way more, like, they outnumber [the negatives]… But no negatives I can think about really, but some pain, but I guess that’s normal, I guess, at first… Well, the thing I thought was actually [more beneficial is] being able to interact with people, not feeling inferior or insecure, I guess. It’s like people have better teeth or perfect teeth, whatever the case may be. That’s the biggest thing for sure*

At the end of the interview, the participant was also asked about his perspective on government dental programs, specifically who should be covered and what treatments should be included. His insights on how such publicly subsidized programs can better serve the population were very enriching. The participant believed that government programs should initially cover low-income individuals and those seeking refugee status in Canada. Additionally, he believed that coverage should be incrementally extended to the middle-income population. Regarding the range of services covered, he believed that comprehensive basic services, such as root canal treatment, cleanings, fillings, and crowns, should be provided at no cost to these targeted populations. Furthermore, he argued that more specialized services, such as orthodontics, should also be included in a public dental program. Ultimately, he believed that incremental policy changes should lead to universal dental coverage and become an integral part of the Canadian healthcare system.

*People coming from abroad, like war-torn places, for example, like asylum seekers, people below a certain amount of income, like $70,000 or $60,000, [should be granted public insurance]… I feel like the things should be covered should be like the very important things root canals, fillings, crowns… Like cleanings, I mean, sure if it’s like possible but definitely the molar damaging things like fillings and RCTs and even braces… I mean the perfect place I feel like, is to be under the umbrella of health care system that everything should be free. But obviously, it will cycle slowly, it should be like the low-income people and, of course, the asylum seekers. Like I said, but in the perfect world, I feel like everyone should have access to it for sure, like other things that are a part of the health care system.*

## 3. Discussion

This case report documents a single patient’s journey through the One Smile Research program, from enrollment to receiving necessary dental care and subsequent oral health follow-ups up to two years post-treatment. Overall, both the clinical and self-reported outcomes could suggest that providing cost-free dental care to individuals facing financial barriers may contribute to improvements in their oral health, psychosocial well-being, and self-esteem, with benefits observed over two years at a minimum.

Qualitative analysis supports the positive effect on overall well-being, highlighting that social confidence gained because of the improvement of his oral and dental health was central to the program’s beneficial impact. Additionally, the analysis provided insights into the patient’s perspective, including the ease of enrollment, despite a three-month wait for registration, as well as the high quality of care received, particularly highlighting the perceived kindness and chair-side manners of the staff. It is important to note that the delay in enrollment occurred because the clinic was operating at full capacity, and we chose not to accept additional patients at the time to avoid delaying care for those already in treatment. This wait time may not reflect the experience of other patients.

The participant believed that the providers’ pleasant approach to dental care fostered greater adherence to and awareness of oral hygiene habits—improvements that were evident after treatment—and proved to be ephemeral. Both the quantitative and qualitative findings indicate better flossing habits up to one year post-treatment; however, subsequent self-reported follow-up data revealed that the patient stopped flossing daily after that point. Similarly, improved brushing habits were sustained for a maximum of 6 months according to the follow-up questionnaires. These findings align with broader studies showing that education and knowledge alone do not lead to sustained behavior change [17]. Importantly, it underscores the value of a top-down approach from the government to continue facilitating access to care, as relying solely on individuals to manage their own health and hygiene may not be sufficient. Although the patient was initially unemployed, he reported gaining part-time employment at both the one-year and two-year follow-ups. Any connection to the dental intervention is anecdotal and cannot be considered causal.

In viewing the quality of care through the lens of the patient’s experience, he reported receiving high-quality treatment. The dental program aligned with the Institute of Medicine’s definition [18], prioritizing “safety” by delivering services through a qualified licensed dentist in accordance with the Royal College of Dental Surgeons of Ontario guidelines [19]. The program’s “effectiveness” was evident in the successful dental treatments provided and ongoing monitoring for positive outcomes. “Patient-centered” care was emphasized through clear communication and the establishment of a treatment plan tailored to the patient’s needs. “Timely” access to dental services addressed long-standing issues, allowing participants to return for emergency treatment even outside the pre-determined follow-up visits, showcasing “efficiency.” The program’s commitment to “equity” was reflected in its availability to everyone, regardless of race, ethnicity, or religion.

The findings of this case relate broadly to other mixed-methods research exploring treatment outcomes and patient experiences among underserved populations. For example, a mixed-methods study from the United Kingdom [20], evaluating a community dental clinic for people experiencing homelessness, demonstrated improvements in accessibility, treatment outcomes, and patient trust linked to compassionate care. Sun et al. [21] examined emergency department visits for nontraumatic dental problems in the United States and identified limited access to preventive dental care as a key factor driving avoidable emergency visits. Their qualitative analysis indicated systemic barriers to care and also highlighted the importance of patient–provider interactions. Together, these studies, along with the present case report, emphasize how addressing both financial and interpersonal aspects is essential to improving dental care access and outcomes for vulnerable populations.

This case report is limited in its evidentiary scope, as it includes only one subject; however, it offers unique insights through an in-depth exploration of the patient’s experiences with the care received and its impact on him. Additionally, this case seeks to understand the patient’s perspective on the coverage of dental care through public programs, which is often evaluated primarily by subject matter experts, administrators, and politicians. The participant’s insights into how publicly funded programs can better serve the population were very valuable. He believes that the government should cover all dental services, including orthodontics, implemented in a staggered approach: starting with low-income individuals, then expanding to middle-income groups, and gradually including everyone. Notably, he expressed the opinion that Canada should ideally have universal dental coverage as part of its healthcare system.

Despite Canada’s universal healthcare, dental care is predominantly privately funded, with only 6% publicly financed [22]. Current government dental programs, administered through provinces and territories, vary significantly and provide limited coverage, typically being restricted to emergency and basic services, and are available only to specific groups, such as children from low-income families, individuals with disabilities, social assistance recipients, and low-income seniors [23]. The new Canadian Dental Care Plan, an income-tested program administered by Health Canada, was recently launched in May 2024 in a phased approach, initially covering seniors, children, and people with disabilities, followed by all individuals from households earning less than $90,000 [24]. This program is expected to cover approximately nine million eligible Canadians when fully implemented. However, those with private insurance are deemed ineligible for the program; for low- and middle-income people, out-of-pocket co-payments create a financial burden, making access to dental care dependent more on cost than on need, which inadvertently exacerbates the inverse care law in Canada [25,26,27]. The results of this case report will hopefully highlight the importance of the continuity of cost-free dental care and may provide insights to the government on why it is important not to have any co-payments for CDCP clients. However, they should not be interpreted as evidence of larger population trends.

To the best of our knowledge, this is the first case report in dentistry in Canada addressing both clinical outcomes and patient perceptions of cost-free dental care, emphasizing the uniqueness of the participant’s experience. While it is essential to acknowledge that these results are specific to one patient and are, therefore, not generalizable, they highlight issues faced by many Canadians, who experience poor oral health and are unable to access dental care due to affordability issues. As such, the findings should be viewed as hypothesis-generating, offering preliminary insights that may inform future research and policy discussions rather than established outcomes.

## 4. Conclusions

The One Smile Research program, a cost-free dental care initiative that provides continuity of care without financial barriers, not only addresses immediate dental needs but also significantly impacts the participant’s sustained oral health, self-esteem, and overall well-being. However, as a single case report, these findings are anecdotal and limited in scope. While they offer insightful information into the potential benefits of reducing financial barriers to dental care, further research with larger populations is needed to draw a generalizable conclusion about the broader implications for universal dental coverage within the framework of Medicare.

## Figures and Tables

**Table 1:** Score ranges and interpretation guidelines.

Validated Scales	Score Ranges	Interpretation
**Psychosocial impact of dental treatment** **(PIDAQ)**	0 to 92	Lower scores indicate a positive impact on psychosocial well-being related to dental treatment.
**Social functioning questionnaire** **(SFQ)**	0 to 24	Lower scores indicate better social functioning.
**The Rosenberg self-esteem scale** **(RSES)**	10 to 40	Higher scores indicate better self-esteem.
**The modified dental anxiety scale** **(MDAS)**	6 to 30	Lower scores indicate less anxiety

## Data Availability

The data used to support the findings of this study are available from the corresponding author upon request.

## Supplementary Materials

The following supporting information can be downloaded at https://www.mdpi.com/article/10.3390/clinpract15080136/s1, File S1: Baseline survey for adults (17+ years old), File S2: Criteria to determine oral health stability for the One Smile Research Program [28], File S3: Follow-up survey for adults (17+ years old), File S4: Qualitative interview.

## Abbreviations

The following abbreviations are used in this manuscript:

PIDAQ	Psychosocial Impact of Dental Aesthetic Questionnaire
MDAS	Modified Dental Anxiety Scale
RSES	Rosenberg Self-Esteem Scale
SFQ	Social Functioning Questionnaire
