# Supplementary material for: A Mixed-Methods Case Report on Oral Health Changes and Patient Perceptions and Experiences Following Treatment at the One Smile Research Program: A 2-Year Follow-Up
Source: Clin Pract. 2025 Jul 23;15(8):136. doi: 10.3390/clinpract15080136 (PMC12384726; doi:10.3390/clinpract15080136)
Supplement: Supplementary file 1 [file clinpract-15-00136-s001.zip › Supplementary file 1_Baseline Survey.pdf]

THE ONE SMILE RESEARCH PROGRAM: BASELINE SURVEY FOR ADULTS (17+ YEARS OLD)

Your Participant ID ___________________________________

(Please write the ID number that was given to you by the Green Shield Canada Clinic).

Thank you for participating in the One Smile Research Program!

This survey will ask you questions about the following:

- Health of your teeth and gums, including any past dental problems, and how this has affected your day-to-day life;
- Your general health and social well-being; and
- Personal information about you, your household, and your financial situation.

If you have any problems or questions, please ask the research assistant for help.

UNIVERSITY OF TORONTO
FACULTY OF DENTISTRY

OneSmile

HEALTH OF TEETH, GUMS AND MOUTH

The following questions ask about the health of your teeth, gums, and mouth and any impacts on your day-to-day life.

Please mark your response in the box with an 'X'

1. In general, would you say the <u>health of your mouth</u> (including your teeth or dentures, tongue, gums, lips, and jaw joints) is…?

    Excellent
    Very good
    Good
    Fair
    Poor

2. How satisfied are you with <u>the appearance of your teeth and/or dentures?</u>

    Very satisfied
    Satisfied
    Neither satisfied or dissatisfied
    Dissatisfied
    Very dissatisfied

3. How much is <u>your day-to-day life</u> affected by the condition of your teeth, lips, jaws, or mouth?

    Not at all
    Very little
    Somewhat
    Moderately
    Very much

4. How much is <u>your overall well-being</u> affected by the condition of your teeth, lips, jaws, or mouth?

    Not at all
    Very little
    Somewhat
    Moderately
    Very much

ORAL HYGIENE PRACTICES

5. How often do you usually brush your teeth and/or dentures?

   Never
   Once a day
   Twice a day
   >Twice a day
   Other (please state how often per day/week/month/year) _________

6. How often do you floss your teeth?

   Never
   Once a day
   Twice a day
   >Twice a day
   Other (please state how often per day/week/month/year) _________

UNIVERSITY OF TORONTO
FACULTY OF DENTISTRY

OneSmile

ORAL HEALTH AND WELL-BEING

7. During the last 12 months, please indicate how often you experienced the following because of problems with your teeth, mouth, or dentures:

Please mark your response in the box with an 'X'

	**Never**	**Hardly ever**	**Occasionally**	**Fairly often**	**Very often**
Have you had trouble pronouncing any words?					
Have you felt that your sense of taste has worsened?					
Have you had painful aching in your mouth?					
Have you found it uncomfortable to eat any foods?					
Have you been self-conscious?					
Have you felt tense?					
Has your diet been unsatisfactory?					
Have you had to interrupt meals?					
Have you found it difficult to relax?					
Have you been a bit embarrassed?					
Have you been a bit irritable with other people?					
Have you had difficulty doing your usual jobs?					
Have you felt that life in general was less satisfying?					
Have you been totally unable to function?					

ORAL HEALTH IMPACT ON FAMILY LIFE

8. During the last 12 months, how often has your oral health caused:

Please mark your response in the box with an 'X'

	**Never**	**Once or twice**	**Sometimes**	**Often**	**Everyday or almost everyday**
Your family activities to be interrupted?					
Disagreement or conflict in the family?					
Financial difficulties for your family?					

DENTAL APPEARANCE

9. Below is a list of statements about your feelings towards your dental appearance.

Please mark your response in the box with an 'X'

	Not at all	A little	Somewhat	Strongly	Very strongly
I am proud of my teeth.					
I like to show my teeth when I smile.					
I am pleased when I see my teeth in the mirror.					
My teeth are attractive to others.					
I am satisfied with the appearance of my teeth.					
I find my tooth position to be very nice.					
I hold myself back when I smile so my teeth don't show so much.					
If I don't know people well I am sometimes concerned what they might think about my teeth.					
I'm afraid other people could make offensive remarks about my teeth.					
I am somewhat inhibited in social contacts because of my teeth.					
I sometimes catch myself holding my hand in front of my mouth to hide my teeth.					
Sometimes I think people are staring at my teeth.					
Remarks about my teeth irritate me even when they are meant jokingly.					
I sometimes worry about what people whom I may be attracted to think about my teeth					
I envy the nice teeth of other people.					
I am somewhat distressed when I see other people's teeth.					
Sometimes I am somewhat unhappy about the appearance of my teeth.					
I think most people I know have nicer teeth than I do.					
I feel bad when I think about what my teeth look like.					
I wish my teeth looked better					
I don't like to see my teeth in the mirror.					
I don't like to see my teeth in photographs.					
I don't like to see my teeth when I look at a video of myself.					

ORAL PAIN

10. Choose a number from 0 to 10 that best describes the pain in your mouth <u>currently</u>.

    If you have no pain, please select zero.

    0 (No pain)
    1
    2
    3
    4
    5
    6
    7
    8
    9
    10 (Pain as bad as it could possibly be)

DENTAL INSURANCE COVERAGE

<u>Please note: Your response to these questions does not affect your ability to receive dental care in this study</u>.

11. Do you have any type of insurance coverage that pays all or part of your dental expenses?

    Yes
    No
    Don't know

    IF YES, is it?

    Please check all that apply.

    Employment-sponsored
    Government-sponsored (e.g., Ontario Works, Ontario Disability Support Program, Ontario Seniors Dental Care Plan)
    Private plan
    Student plan
    Don't know

UNIVERSITY OF TORONTO
FACULTY OF DENTISTRY

OneSmile

DENTAL CARE AFFORDABILITY/PAST COST BARRIERS TO DENTAL CARE

The following questions will help us understand your dental treatment needs in the last two years, and any associated challenges in receiving care.

Please note: Your response to these questions does not affect your ability to receive dental care in this study.

You had previously indicated that you could not afford all the dental care that you needed in the last two years.

12. What dental treatment did you need?

    Please check all that apply.

    - Check-up
    - X-rays
    - Dental hygiene appointment
    - Fillings
    - Tooth extraction
    - Root canal
    - Crown/cap/bridge
    - Dentures
    - Orthodontic/braces
    - Other, please describe: ________________________
    - Don't know
    - Not applicable (I have not visited a dentist in the last 2 years) (***skip to next section: Dental Anxiety)***

13. Did the dentist or dental office tell you how much your treatment would cost?

    - Yes
    - No
    - Don't know
    - Not applicable

    IF YES, approximately how much was the cost? _________________ CAD

    IF NO, approximately how much do you think it would have cost?

    ______________ CAD

14. Approximately how much of the treatment costs would be covered by your insurance?

    You can answer in how much ($) would be covered or the percent (%) of the treatment cost that would be covered.

    _____________ (CAD / %) (please circle one)

15. Approximately how much money would you have been able to pay out-of-pocket for the dental treatment? ___________ CAD

DENTAL ANXIETY

16. In terms of past dental visits, how anxious do you get, if at all, with the dental visit?

Please mark your response in the box with an 'X'

	**Not anxious**	**Slightly anxious**	**Fairly anxious**	**Very anxious**	**Extremely anxious**
If you went to your dentist for treatment tomorrow, how would you feel?					
If you were sitting in the waiting room (waiting for treatment), how would you feel?					
If you were about to have a tooth drilled, how would you feel?					
If you were about to have your teeth scaled and polished, how would you feel?					
If you were about to have a local anesthetic injection in your gum, how would you feel?					
To what extent are you anxious about the cost of the dental treatment when you go to the dentist?					

UNIVERSITY OF TORONTO
FACULTY OF DENTISTRY

OneSmile

OVERALL HEALTH AND WELL-BEING

17. In general, would you say your health is...?

    Excellent
    Very good
    Good
    Fair
    Poor

18. Thinking about the amount of stress in your life, would you say that most of your days are…?

    Not at all stressful
    Not very stressful
    A bit stressful
    Quite a bit stressful
    Extremely stressful

19. Under each heading, please select the ONE box that best describes your health TODAY.

Mobility:

I have no problems in walking about
I have slight problems in walking about
I have moderate problems in walking about
I have severe problems in walking about
I am unable to walk about

Looking after oneself:

I have no problems washing or dressing myself
I have slight problems washing or dressing myself
I have moderate problems washing or dressing myself
I have severe problems washing or dressing myself
I am unable to wash or dress myself

Doing usual activities:
(e.g. work, study, housework, family or leisure activities)

I have no problems doing my usual activities
I have slight problems doing my usual activities
I have moderate problems doing my usual activities
I have severe problems doing my usual activities
I am unable to do my usual activities

Pain and discomfort:

I have no pain or discomfort
I have slight pain or discomfort
I have moderate pain or discomfort
I have severe pain or discomfort
I have extreme pain or discomfort

Feeling worried, sad or unhappy:

I am not anxious or depressed
I am slightly anxious or depressed
I am moderately anxious or depressed
I am severely anxious or depressed
I am extremely anxious or depressed

20. We would like to know how good or bad your health is TODAY.

This scale is numbered from 0 to 100.

- 100 means the best health you can imagine
- 0 means the worst health you can imagine.

Place a mark ‘**X**’ on the scale below to indicate your health today.

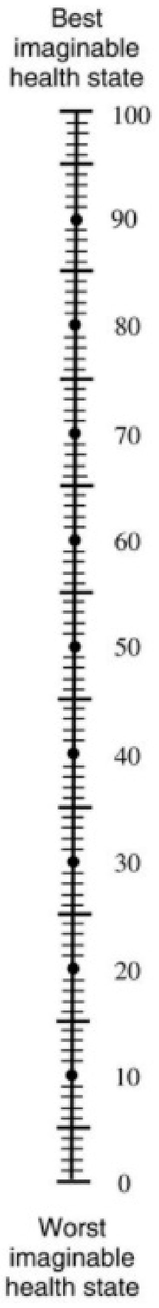

Now, please write the number you marked on the scale in the box.

UNIVERSITY OF TORONTO
FACULTY OF DENTISTRY

OneSmile

OVERALL HEALTH AND WELL-BEING

21. Below is a list of statements dealing with your general feelings about yourself.

Please mark your response in the box with an 'X'

	**Strongly agree**	**Agree**	**Disagree**	**Strongly disagree**
On the whole, I am satisfied with myself.				
At times I think I am no good at all.				
I feel that I have a number of good qualities.				
I am able to do things as well as most other people.				
I feel I do not have much to be proud of.				
I certainly feel useless at times.				
I feel that I'm a person of worth, at least on an equal plane with others.				
I wish I could have more respect for myself.				
All in all, I am inclined to feel that I am a failure.				
I take a positive attitude toward myself.				

UNIVERSITY OF TORONTO
FACULTY OF DENTISTRY

OneSmile

SOCIAL WELL-BEING

22. Please read the statements below and select the response that reflects how you have been feeling recently:

I complete my tasks at work and home satisfactorily.

- Most of the time
- Quite often
- Sometimes
- Not at all

I find my tasks at work and at home very stressful.

- Most of the time
- Quite often
- Sometimes
- Not at all

I have no money problems.

- No problems at all
- Slight worries only
- Definite problems
- Very severe problems

I have difficulties in getting and keeping close relationships.

- Severe difficulties
- Some problems
- Occasional problems
- No problems at all

I have problems in my sex life.

- Severe problems
- Moderate problems
- Occasional problems
- No problems at all
- Not applicable

I get on well with my family and other relatives.

- Yes, definitely
- Yes, usually
- No, some problems
- No, severe problems

I feel lonely and isolated from other people.

Almost all the time
Much of the time
Not usually
Not at all

I enjoy my spare time

Very much
Sometimes
Not often
Not at all

SOCIO-DEMOGRAPHIC INFORMATION
This is the last section of the survey.

Personal factors affect health in different ways, which we need to know when conducting this research; therefore, the following questions relate to some personal information about you and your family.

Please note no information you share will be disclosed to anyone at any point in time.

23. Year of birth ______________________________ (YYYY)

24. What was your sex assigned at birth?

Male
Female
Intersex
Prefer not to answer

25. Your gender may be different from your sex assigned at birth and may be different from what is indicated on your legal documents.

What is your gender?

Man
Woman
Non-binary
Prefer to describe:________________
Prefer not to answer

26. We know that people of different races do not have significantly different genetics. But our race still has important social consequences.

    Which race category/categories best describes you?

    Please check all that apply.

    Black (e.g., African, Afro-Caribbean, African Canadian descent)
    East/Southeast Asian (e.g., Chinese, Korean, Japanese, Taiwanese descent or Filipino, Vietnamese, Cambodian, Thai, Indonesian, other Southeast Asian descent)
    Indigenous (First Nations, Metis, Inuk/Inuit descent)
    Latino (e.g., Latin American, Hispanic decent)
    Middle Eastern (e.g., Arab, Persian, West Asian descent, Afghan, Egyptian, Iranian, Lebanese, Turkish, Kurdish)
    South Asian (e.g., Indian, Pakistani, Bangladeshi, Sri Lankan, Indo-Caribbean)
    White (e.g., European descent)
    Other race category (e.g., includes values not described above), please describe:_____________
    Prefer not to answer
    Don't know

27. Were you born in Canada?
    Yes ***(skip to Question 46)***
    No
    Prefer not to answer (skip to question in what year did you move to Canada)
    Don't know

    IF NO (to question 45), what country were you born in? ---------------

    IF NO (to question 45), in what year did you move to Canada?

    _____________(YYYY)

MARITAL STATUS AND LIVING ARRANGEMENT

28. What is your marital status?

    Married/common-law
    Widowed
    Separated/divorced
    Single, never married
    Prefer not to answer

29. How many people live in your household (including yourself)?

    1
    2

3
4
5 or more
Prefer not to answer

30. Is this household ...

Owned by you or a member of this household? (even if the mortgage is still being paid)
Rented (even if no cash rent is paid)
Prefer not to answer
Don't know

EDUCATION AND EMPLOYMENT STATUS

31. What is the highest certificate, diploma, or degree that you have completed?

    Less than a high school diploma
    High school diploma or a high school equivalency certificate
    Trade certificate or diploma
    College, CEGEP, or other non-university certificate or diploma
    University certificate or diploma below the bachelor's level
    Bachelor's degree
    University certificate, diploma, or a degree above the bachelor's level
    Prefer not to answer

32. Do you have a paid job, either full-time or part-time, including salaried and/or self-employment?

    Yes
    No
    Prefer not to answer

33. Which of the choices best describes your current employment situation?

    Working - Full-time (at least 32 hours per week)
    Working - Part-time (less than 32 hours per week)
    Working at reduced hours due to COVID-19
    On leave but still employed
    Temporarily/permanently laid off due to COVID-19
    Temporarily/permanently laid off unrelated to COVID-19
    Unemployed and looking for work
    Wanting to work, but unemployed due to a health-related reason
    Being a homemaker
    Retired
    Receiving/awaiting approval for disability payments
    Not currently employed
    Full-time student
    Other, please describe: ________________________
    Prefer not to answer

INCOME INFORMATION

How much money we have can make a big difference in our lives. We appreciate you answering the following questions about your family's financial situation as accurately as you can, remembering that this information is confidential and will not be associated with your name.

Again, your information will not be shared nor affect your enrollment in the study.

34. Can you estimate in which of the following categories your family's usual annual household income falls (before income tax deduction, from all sources of income)?

    Less than $5,000
    $5,000 to less than $10,000
    $10,000 to less than $15,000
    $15,000 to less than $20,000
    $20,000 to less than $30,000
    $30,000 to less than $40,000
    $40,000 to less than $50,000
    $50,000 to less than $60,000
    $60,000 to less than $70,000
    $70,000 to less than $80,000
    $80,000 to less than $90,000
    $90,000 to less than $100,000
    $100,000 to less than $150,000
    $150,000 and over
    Prefer not to answer
    Don't know

35. What is your best estimate of your family's total annual household income received by all household members, from all sources, before taxes and deductions, during the last year?

    Note: Income can come from various sources such as from work, investments, pensions or government. Examples include Employment Insurance, Social Assistance, Child Tax Benefit and other income such as child support, spousal support (alimony) and rental income.

    ____________________ (Min 0 to Max 1,000,000 CAD)

36. Was your family's annual household income reduced in 2020 because of COVID-19?

Yes
No
Prefer not to answer
Don't know

37. What is your best estimate of your total personal annual income received, from all sources, before taxes and deductions, during the last year?

__________________ (Min 0 to Max 1,000,000 CAD)

END OF SURVEY

You are now at the end of the survey!

If you would like to return to the survey to review your responses, you could so, otherwise, please give the research associate your completed survey.
