# Supplementary material for: A Mixed-Methods Case Report on Oral Health Changes and Patient Perceptions and Experiences Following Treatment at the One Smile Research Program: A 2-Year Follow-Up
Source: Clin Pract. 2025 Jul 23;15(8):136. doi: 10.3390/clinpract15080136 (PMC12384726; doi:10.3390/clinpract15080136)
Supplement: Supplementary file 1 [file clinpract-15-00136-s001.zip › Supplementary file 2_Oral health stability criteria.pdf]

Criteria to determine oral health stability for the One Smile Research Program

<u>In general, a participant has reached oral health stability if:</u>

a. They are not experiencing any oral pain and/or infection;
b. They have had their immediate periodontal needs addressed; and
c. They require no immediate restorative, endodontic, and/or surgical treatment.

**Category**	**Criteria**
Pain	• Individual is free of pain, specific to the oral cavity; does not include gum tenderness resulting from treatment/oral self-care.
Soft tissue	• There are no suspicious lesions that require evaluation or biopsy. Note: Benign oral lesions may be present that require monitoring.
Radiography	• Current dental radiographs are free from suspicious lesions that require evaluation or biopsy<br>• Benign radiographic lesions may be present that require monitoring
Dental caries	• There is no evidence of active (progressing) caries extending into the dentin.<br>• Active (progressing) caries are limited to enamel and preventive care is indicated, and tooth is monitored.<br>• Arrested caries or no evident cavitation; preventive care is indicated, and tooth is monitored.
Pulp and periapical tissues	• There are no teeth with signs and symptoms of irreversible pulpal damage or necrotic pulp.<br>• There are no teeth with incomplete endodontic treatment (in other words, RCT has been completed), which has been initiated.<br>• Asymptomatic previous endodontic/periapical lesions or incomplete/inadequate endodontic treatment or condition that patient does not want interference with; tooth is monitored.
Periodontal diseases	• There is no evidence of active periodontal disease that is beyond control by primary self-care. Professional care in the form of maintenance therapy may be required as a secondary measure to manage the patient's condition.<br>• There are no periodontally involved teeth with associated apical involvement, which are untreated, and when treated, do not show both clinical and radiographic signs of resolution.<br>• Asymptomatic radiographic bone loss that patient does not want interference with; preventive care is provided and condition is monitored.<br>• Patient is not willing to go for suggested advanced periodontal treatment. Condition is monitored.
Restorations	• There are no defective permanent restorations (cracked, loose, or leaking) that are causing symptoms or tissue damage or cannot be maintained by the patient.<br>• There are no posterior teeth requiring immediate protective cuspal coverage to maintain the structural integrity of the tooth. Condition would be monitored. Crown may be required at a later date.<br>• Chipped or fractured tooth (missing tooth material, e.g., abrasion, attrition, erosion, abfraction, minor fracture, minor chipping), or restorative material not needing replacement.

Teeth and Roots	• There are no unerupted, partially erupted, or malposed teeth with (historical), clinical, or radiographic signs or symptoms of pathosis that require extraction.<br>• There are no symptomatic non-restorable teeth.<br>• There are no functionless roots in communication with the oral cavity. Note: Buried roots with no associated pathology or endodontically treated roots may be left in situ and monitored.<br>• If patient denies extraction with informed consent; would be monitored.
Third Molars	• There are no unerupted, partially erupted, or malposed third molars with (historical), clinical, or radiographic signs or symptoms of pathosis that require extraction.<br>• If patient denies extraction/treatment with informed consent; would be monitored.

Adapted from the Canadian Forces Dental Care Program criteria [28] for dental fitness classification 1 and 2.

Reference

28. Groves, R.R. Dental Fitness Classification in the Canadian Forces. Mil Med. **2008** Jan 1;173(suppl_1):18–22. Doi: https://doi.org/10.7205/milmed.173.supplement_1.18
