# Supplementary material for: A Mixed-Methods Case Report on Oral Health Changes and Patient Perceptions and Experiences Following Treatment at the One Smile Research Program: A 2-Year Follow-Up
Source: Clin Pract. 2025 Jul 23;15(8):136. doi: 10.3390/clinpract15080136 (PMC12384726; doi:10.3390/clinpract15080136)
Supplement: Supplementary file 1 [file clinpract-15-00136-s001.zip › Supplementary file 3_Follow-up Survey.pdf]

UNIVERSITY OF TORONTO
FACULTY OF DENTISTRY

OneSmile

THE ONE SMILE RESEARCH PROGRAM: FOLLOW-UP SURVEY FOR ADULTS (17+ YEARS OLD)

Your Participant ID ___________________________________

(Please write the ID number that was given to you by the Green Shield Canada Clinic).

Thank you for participating in the One Smile Research Program!

This survey will ask you some of the same questions you were asked before you received dental treatment and some additional ones on how your oral health and general health have changed since receiving treatment.

If you have any problems or questions please ask the research assistant for help.

UNIVERSITY OF TORONTO
FACULTY OF DENTISTRY

OneSmile

HEALTH OF TEETH, GUMS AND MOUTH

The following questions ask about the health of your teeth, gums, and mouth and any impacts on your day-to-day life.

Please mark your response in the box with an 'X'

1. In general, would you say the health of your mouth (including your teeth or dentures, tongue, gums, lips, and jaw joints) is…?

   Excellent
   Very good
   Good
   Fair
   Poor

2. How satisfied are you with the appearance of your teeth and/or denture?

   Very satisfied
   Satisfied
   Neither satisfied or dissatisfied
   Dissatisfied
   Very dissatisfied

3. How much is your day-to-day life affected by the condition of your teeth, lips, jaws, or mouth?

   Not at all
   Very little
   Somewhat
   Moderately
   Very much

4. How much is your overall well-being affected by the condition of your teeth, lips, jaws, or mouth?

   Not at all
   Very little
   Somewhat
   Moderately
   Very much

5. Has there been a change in your oral health status since you last completed the survey?

Yes
No
Don’t know

6. How would you describe your oral health status now compared to when you last completed the survey? *Please **CIRCLE** a number.*

-7	-6	-5	-4	-3	-2	-1	0	1	2	3	4	5	6	7
A very great deal worse							Unchanged							A very great deal better

ORAL HYGIENE PRACTICES

7. How often do you usually brush your teeth and/or dentures?

    Never
    Once a day
    Twice a day
    >Twice a day
    Other (please state how often per day/week/month/year) __________

8. How often do you floss your teeth?

    Never
    Once a day
    Twice a day
    >Twice a day
    Other (please state how often per day/week/month/year)) __________

ORAL HEALTH AND WELL-BEING

9. Since you last completed this survey, please indicate how often you experienced the following because of problems with your teeth, mouth, or dentures:

Please mark your response in the box with an 'X'

	Never	Hardly ever	Occasionally	Fairly often	Very often
Have you had trouble pronouncing any words?					
Have you felt that your sense of taste has worsened?					
Have you had painful aching in your mouth?					
Have you found it uncomfortable to eat any foods?					
Have you been self-conscious?					
Have you felt tense?					
Has your diet been unsatisfactory?					
Have you had to interrupt meals?					
Have you found it difficult to relax?					
Have you been a bit embarrassed?					
Have you been a bit irritable with other people?					
Have you had difficulty doing your usual jobs?					
Have you felt that life in general was less satisfying?					
Have you been totally unable to function?					

ORAL HEALTH IMPACT ON FAMILY LIFE

10. Since you last completed this survey, how often has your oral health caused:

Please mark your response in the box with an 'X'

	**Never**	**Once or twice**	**Sometimes**	**Often**	**Everyday or almost everyday**
Your family activities to be interrupted?					
Disagreement or conflict in the family?					
Financial difficulties for your family?					

DENTAL APPEARANCE

11. Below is a list of statements about your feelings towards your dental appearance.

Please mark your response in the box with an 'X'

	**Not at all**	**A little**	**Somewhat**	**Strongly**	**Very strongly**
I am proud of my teeth.					
I like to show my teeth when I smile.					
I am pleased when I see my teeth in the mirror.					
My teeth are attractive to others.					
I am satisfied with the appearance of my teeth.					
I find my tooth position to be very nice.					
I hold myself back when I smile so my teeth don't show so much.					
If I don't know people well I am sometimes concerned what they might think about my teeth.					
I'm afraid other people could make offensive remarks about my teeth.					
I am somewhat inhibited in social contacts because of my teeth.					
I sometimes catch myself holding my hand in front of my mouth to hide my teeth.					
Sometimes I think people are staring at my teeth.					
Remarks about my teeth irritate me even when they are meant jokingly.					
I sometimes worry about what people whom I may be attracted to think about my teeth					
I envy the nice teeth of other people.					
I am somewhat distressed when I see other people's teeth.					
Sometimes I am somewhat unhappy about the appearance of my teeth.					
I think most people I know have nicer teeth than I do.					
I feel bad when I think about what my teeth look like.					
I don't like to see my teeth in the mirror.					
I don't like to see my teeth in photographs.					
I don't like to see my teeth when I look at a video of myself.					

ORAL PAIN

12. Choose a number from 0 to 10 that best describes the pain in your mouth currently.

    If you have no pain, please select zero.

    0 (No pain)
    1
    2
    3
    4
    5
    6
    7
    8
    9
    10 (Pain as bad as it could possibly be)

DENTAL INSURANCE COVERAGE

Please note: Your response to these questions does not affect your ability to receive dental care in this study.

13. Has there been any changes to your dental insurance coverage since you last completed the survey?

    Yes
    No
    Don't know

    IF YES, what type of dental insurance coverage do you have?

    Please check all that apply.

    Employment-sponsored
    Government-sponsored (for example, Health Smiles Ontario)
    Private plan
    Student plan
    I no longer have insurance coverage
    Don't know

DENTAL ANXIETY

24. In terms of past dental visits, how anxious do you get, if at all, with the dental visit?

Please mark your response in the box with an 'X'

	**Not anxious**	**Slightly anxious**	**Fairly anxious**	**Very anxious**	**Extremely anxious**
If you went to your dentist for treatment tomorrow, how would you feel?					
If you were sitting in the waiting room (waiting for treatment), how would you feel?					
If you were about to have a tooth drilled, how would you feel?					
If you were about to have your teeth scaled and polished, how would you feel?					
If you were about to have a local anesthetic injection in your gum, how would you feel?					
To what extent are you anxious about the cost of the dental treatment when you go to the dentist?					

OVERALL HEALTH AND WELL-BEING

25. In general, would you say your health is...?

    Excellent
    Very good
    Good
    Fair
    Poor

26. Thinking about the amount of stress in your life, would you say that most of your days are…?

    Not at all stressful
    Not very stressful
    A bit stressful
    Quite a bit stressful
    Extremely stressful

27. Under each heading, please select the ONE box that best describes your health TODAY.

Mobility:

I have no problems in walking about
I have slight problems in walking about
I have moderate problems in walking about
I have severe problems in walking about
I am unable to walk about

Looking after oneself:

I have no problems washing or dressing myself
I have slight problems washing or dressing myself
I have moderate problems washing or dressing myself
I have severe problems washing or dressing myself
I am unable to wash or dress myself

Doing usual activities:
(e.g. work, study, housework, family or leisure activities)

I have no problems doing my usual activities
I have slight problems doing my usual activities
I have moderate problems doing my usual activities
I have severe problems doing my usual activities
I am unable to do my usual activities

Pain and discomfort:

I have no pain or discomfort
I have slight pain or discomfort
I have moderate pain or discomfort
I have severe pain or discomfort
I have extreme pain or discomfort

Feeling worried, sad or unhappy:

I am not anxious or depressed
I am slightly anxious or depressed
I am moderately anxious or depressed
I am severely anxious or depressed
I am extremely anxious or depressed

28. We would like to know how good or bad your health is TODAY.

This scale is numbered from 0 to 100.

- 100 means the best health you can imagine
- 0 means the worst health you can imagine.

Place a mark ‘**X**’ on the scale below to indicate your health today.

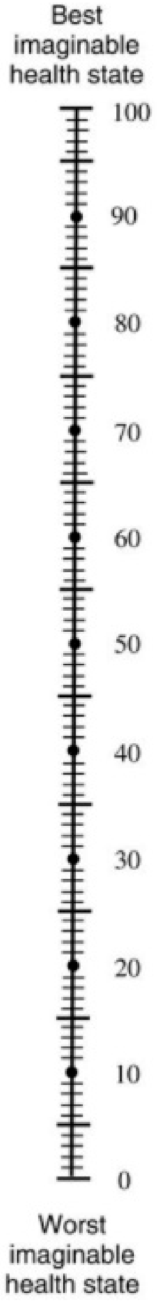

Now, please write the number you marked on the scale in the box.

OVERALL HEALTH AND WELL-BEING

29. Below is a list of statements dealing with your general feelings about yourself.

Please mark your response in the box with an 'X'

	Strongly agree	Agree	Disagree	Strongly disagree
On the whole, I am satisfied with myself.				
At times I think I am no good at all.				
I feel that I have a number of good qualities.				
I am able to do things as well as most other people.				
I feel I do not have much to be proud of.				
I certainly feel useless at times.				
I feel that I'm a person of worth, at least on an equal plane with others.				
I wish I could have more respect for myself.				
All in all, I am inclined to feel that I am a failure.				
I take a positive attitude toward myself.				

SOCIAL WELL-BEING

30. Please read the statements below and select the response that reflects how you have been feeling recently:

I complete my tasks at work and home satisfactorily.

Most of the time
Quite often
Sometimes
Not at all

I find my tasks at work and at home very stressful.

Most of the time
Quite often
Sometimes
Not at all

I have no money problems.

No problems at all
Slight worries only
Definite problems
Very severe problems

I have difficulties in getting and keeping close relationships.

Severe difficulties
Some problems
Occasional problems
No problems at all

I have problems in my sex life.

Severe problems
Moderate problems
Occasional problems
No problems at all
Not applicable

I get on well with my family and other relatives.

Yes, definitely
Yes, usually
No, some problems
No, severe problems

I feel lonely and isolated from other people.

Almost all the time
Much of the time
Not usually
Not at all

I enjoy my spare time

Very much
Sometimes
Not often
Not at all

SOCIO-DEMOGRAPHIC INFORMATION

This is the last section of the survey.

Personal factors affect health in different ways, which we need to know when conducting this research; therefore, the following questions relate to some personal information about you and your family.

Please note no information you share will be disclosed to anyone at any point in time.

31. Has there been any changes in your marital status since you last completed the survey?

    Yes
    No
    Prefer not to answer

    IF YES, which of the choices best describes your current marital status?

    Married/common-law
    Widowed
    Separated/divorced
    Single, never married
    Prefer not to answer

32. Has there been any change to the number of people living in your household (including yourself) since you last completed the survey?

    Yes
    No
    Prefer not to answer

    IF YES, how many people currently live in your household (including yourself)?

    1
    2
    3
    4
    5 or more
    Prefer not to answer

33. Has there been any changes to your home ownership since you last completed the survey?

Yes
No
Prefer not to answer

IF YES, which of the choices best describes your current home ownership?

Owned by you or a member of this household (even if the mortgage is still being paid)
Rented (even if no cash rent is paid)
Prefer not to answer
Don't know

34. Has there been any changes to your education since you last completed the survey?

Yes
No
Prefer not to answer

IF YES, what is the highest certificate, diploma, or degree that you have completed?

Less than a high school diploma
High school diploma or a high school equivalency certificate
Trade certificate or diploma
College, CEGEP, or other non-university certificate or diploma
University certificate or diploma below the bachelor's level
Bachelor's degree
University certificate, diploma, or a degree above the bachelor's level
Prefer not to answer

35. Have there been any changes in your employment status since you last completed the survey?

Yes
No
Prefer not to answer

IF YES, which of the choices best describes your current employment situation?

Working - Full-time (at least 32 hours per week)
Working - Part-time (less than 32 hours per week)
Working at reduced hours due to COVID-19
On leave but still employed

Temporarily laid off due to COVID-19
Temporarily laid off unrelated to COVID-19
Unemployed and looking for work
Wanting to work, but unemployed due to a health-related reason
Being a homemaker
Retired
Receiving/awaiting approval for disability payments
Not currently employed
Full-time student
Other, please describe: ________________________
Prefer not to answer

INCOME INFORMATION

How much money we have can make a big difference in our lives. We appreciate you answering the following questions about your family's financial situation as accurately as you can, remembering that this information is confidential and will not be associated with your name.

Again, your information will not be shared nor affect your enrollment in the study.

36. Have there been any changes in your family's household income since you last completed the survey?

    Yes (***answer Question 43, 44, and 45***)
    No (***skip to next section: Financial Situation***)
    Prefer not to answer

37. Can you estimate in which of the following categories your family's usual annual household income falls (before income tax deduction, from all sources of income)?

    Less than $5,000
    $5,000 to less than $10,000
    $10,000 to less than $15,000
    $15,000 to less than $20,000
    $20,000 to less than $30,000
    $30,000 to less than $40,000
    $40,000 to less than $50,000
    $50,000 to less than $60,000
    $60,000 to less than $70,000
    $70,000 to less than $80,000
    $80,000 to less than $90,000
    $90,000 to less than $100,000
    $100,000 to less than $150,000
    $150,000 and over
    Prefer not to answer
    Don't know

38. What is your best estimate of your total family annual household income received by all household members, from all sources, before taxes and deductions?

Income can come from various sources such as from work, investments, pensions or government. Examples include Employment Insurance, Social Assistance, Child Tax Benefit and other income such as child support, spousal support (alimony) and rental income.

____________________ (Min 0 to Max 1,000,000 CAD)

39. What is your best estimate of your total personal annual income received, from all sources, before taxes and deductions?

__________________ (Min 0 to Max 1,000,000 CAD)

END OF SURVEY

You are now at the end of the survey!

If you would like to return to the survey to review your responses, you could so, otherwise, please give the research associate your completed survey.
