# Supplementary material for: A Mixed-Methods Case Report on Oral Health Changes and Patient Perceptions and Experiences Following Treatment at the One Smile Research Program: A 2-Year Follow-Up
Source: Clin Pract. 2025 Jul 23;15(8):136. doi: 10.3390/clinpract15080136 (PMC12384726; doi:10.3390/clinpract15080136)
Supplement: Supplementary file 1 [file clinpract-15-00136-s001.zip › Supplementary file 4_qualitative interview.pdf]

Qualitative Interview Guide

The interviewer will:

i) greet the patient and confirm their identity, and introduce themself.

ii) review the purpose of the study and obtain informed consent to interview the patient.

iii) remind the patient that the contents of the interview will be kept confidential and data will be reported in aggregate so as not to disclose patient identity.

Questions

1. Can you tell me a bit about yourself?
   (Focusing on your oral health prior to becoming a participant and your previous dental visits).
2. How did you come to know about the program?
3. Do you think the enrolment process was an easy and comfortable experience?
4. How was your experience with the research team? Was the team able to help you understand your role and responsibilities as a participant?
5. How was your experience with the clinical team? Was the team able to understand your problems and were your treatment needs addressed?
6. Has receiving treatment as a participant in the One Smile Program impacted your oral health care behavior in any way?
7. Has receiving treatment as a participant in the One Smile Program impacted your life in any way (include, social, financial and employment related impacts)? If yes, would be great if you please share.
8. Is there anything in the program that did not meet your expectations or you hope could have been done differently?
9. In the future when this program concludes, would you be visiting a dentist routinely?
10. What are your views about dental clinics who provide free dental care?
11. In the future if we have a government dental program, should all dental procedures be available for a few people or some procedures available for everyone?
12. If you feel all procedures should be available for a few people, who do you think this group of people should be?
13. If you feel some procedures should be available for everyone, what procedures do you feel should be included?
